# Immune checkpoints HLA-E:CD94-NKG2A and HLA-C:KIR2DL1 complementarily shield circulating tumor cells from NK-mediated immune surveillance

**DOI:** 10.1038/s41421-024-00646-3

**Published:** 2024-02-09

**Authors:** Xiaowei Liu, Fengli Zuo, Jinen Song, Leyi Tang, Xueyan Wang, Xinyu Liu, Hao Zhang, Zhankun Yang, Jing Jing, Xuelei Ma, Hubing Shi

**Affiliations:** 1 Institute for Breast Health Medicine, State Key Laboratory of Biotherapy, West China Hospital, Sichuan University, Chengdu, Sichuan China; 2 Department of Pancreatic Surgery, West China Hospital, Sichuan University, Chengdu, Sichuan China; 3 College of Chemical Engineering, Shijiazhuang University, Shijiazhuang, Hebei China; 4 Department of Biotherapy, West China Hospital and State Key Laboratory of Biotherapy, Sichuan University, Chengdu, Sichuan China

**Keywords:** Immunosurveillance, Metastasis

Dear Editor,

Tumor metastasis is the leading cause of cancer-related deaths^1^. Circulating tumor cells (CTCs), shed from the primary tumor, play a “seed” role in initiating the formation of metastatic lesions^2^. Identifying immune checkpoints on CTCs may provide novel immunotherapy strategies to prevent tumor metastasis. Recently, we have unraveled that natural killer cells (NKs) play a predominant role in immune surveillance on CTCs, and CTCs escape the surveillance by engaging an immune checkpoint HLA-E:CD94-NKG2A^3^. Blockade of this immune checkpoint significantly prevents tumor metastasis by eliminating CTCs. Our results provide a potential strategy to prevent CTC-mediated tumor metastasis by disrupting the immune checkpoint between CTCs and NKs. However, due to the diversity of NKs^4,5^, many researchers raise a question of whether or not other immune checkpoints also facilitate the escape of immune surveillance in addition to the reported molecular pair^6^. Identifying the immune checkpoint molecules between CTCs and other subtypes of NKs may provide a comprehensive strategy for prevention of tumor metastasis by activating NK-mediated CTCs elimination.

To this end, we profiled the single-cell transcriptomes of primary tumors, CTC circulations, and metastatic lesions from six patients with pancreatic ductal adenocarcinoma (PDAC)^3,7^. A total of 74,206 cells, including 523 CTCs, are presented by *t*-SNE plot based on the cell type (Fig. 1a), patient, and tissue origin (Supplementary Fig. S1a–d). According to canonical markers, these cells are categorized into 4 kinds of non-immunocytes, namely epithelial cells, fibroblasts, endothelial cells, and CTCs; and 15 immunocytes subtypes, including NKs, NK-T cells (NKT), CD8 exhausted T-cells (CD8 Ex), CD8 effector T-cells (CD8 EFF), memory T-cells, naïve T-cells, Treg cells, B cells, M1 macrophages, M2 macrophages, classical DCs (cDC), plasma DCs (pDC), neutrophils, monocytes, and mast cells (Supplementary Fig. S1e, f). In addition, the malignant cells (tumor cells) are distinguished from normal epithelial cells by CopyKAT^3^.

**Fig. 1:**
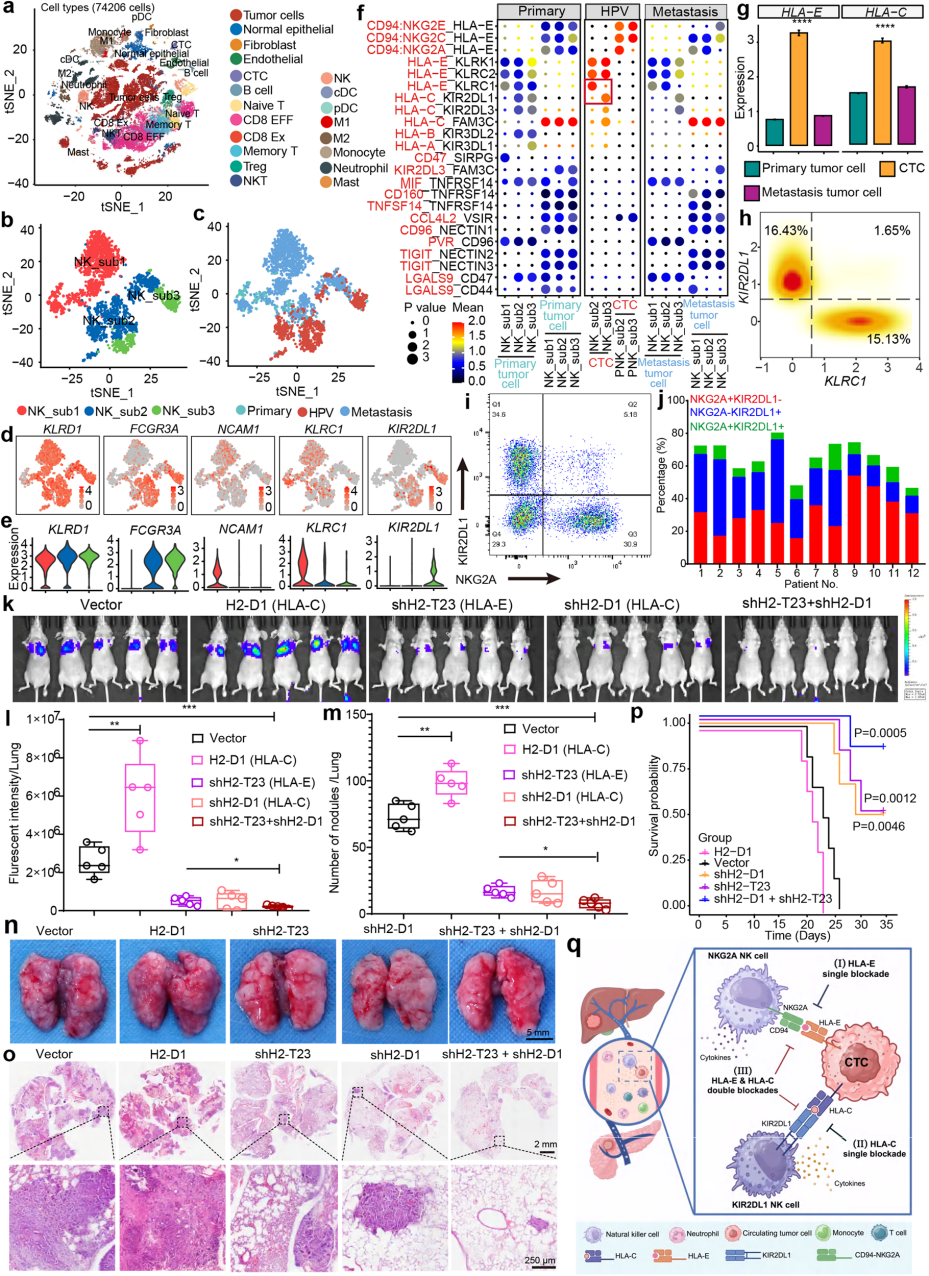
Complementary immune checkpoints HLA-E:CD94-NKG2A and HLA-C:KIR2DL1 between CTCs and NKs. **a** Cell type characterization of primary, circulating (hepatic portal vein, HPV), and metastatic lesions from PDACs (*n* = 18 samples, 74,206 cells). **b**, **c** Clustering of NK cells by cell type (**b**) and tissue origin (**c**). **d**, **e** The levels of indicated marker genes in each subtype of NKs are presented by t-SNE plots (**d**) and quantified by violin plots (**e**). **f** The immune checkpoint molecules between tumor cells/CTCs and NKs in each corresponding tissue origin were analyzed by CellPhoneDB (version 2.0). The color represents the mean expression of ligand–receptor pairs, while the dot size indicates statistical significance. **g** The expression of *HLA-E* and *HLA-C* on tumor cells from indicated tissues. Mean ± SE, two sides Wilcoxon test, ****P < 0.0001. **h** Multivariate kernel density plots depict the bimodal distribution of *KLRC1* (NKG2A) and *KIR2DL1* on NKs of HPV blood. **i** NKG2A^+^ and KIR2DL1^+^ NKs from the blood of PDAC patient 1 are presented. **j** The proportion of NKG2A^+^ and KIR2DL1^+^ NKs from the blood of PDAC patients were quantified by flow cytometry. **k**, **l** Tumor cells metastasizing to the lung were visualized (**k**) and quantified (**l**) by bioluminescence imaging system 15 days after intravenous injection (i.v.*)* inoculation of H2-T23 (HLA-E) or/and H2-D1 (HLA-C) pre-knockdown KPC-Luc cells. **m**, **n** The metastatic tumor nodules in the lung were counted (**m**), and representative images were displayed (**n**). *P < 0.05; **P < 0.01; ***P < 0.001, *t*-test. **o** Pathological aberrancy of lungs from (**n**) was evaluated by H&E staining. **p** Kaplan–Meier plot shows the overall survival of mice (*n* = 5) with indicated treatments. **q** Schematic diagram of complementary immune checkpoints HLA-E:CD94-NKG2A- and HLA-C:KIR2DL1-mediated evasion of CTCs from NKs immune surveillance.

To investigate the immune function of NKs, we re-clustered NKs from primary tumors, blood circulation, and metastatic lesions. Based on the canonical functional markers^3,5^, these NKs are categorized into three sub-groups, namely sub 1 (*FCGR3A*^*−*^*NCAM1*^hi^*KLRC1*^+^), sub 2 (*FCGR3A*^+^*NCAM1*^low^*KLRC1*^+^), and sub 3 (*FCGR3A*^+^*NCAM1*^low^*KLRC1*^*−*^) (Fig. 1b–e). The sub 1, which is mainly observed in solid lesions, is defined as a population with strong characters of cell cycle, cytokine production, and up-regulated metabolisms, including glycolysis gluconeogenesis and fatty acid metabolism (Supplementary Fig. S2a, b). Consistently, the expression level of cell cycle (*FOS, FOSB, JUN, CEBPD*), cytokines (*GZMK, XCL1, XCL2, CCL3*) and metabolism-related genes (*CD160, GSTP1, AREG, ALOX5AP*) in sub 1, are higher than those in sub 2/3 (Supplementary Fig. S3a, b). Moreover, sub 1 has a relatively higher proliferation score, indicating that sub 1 has a proliferation ability (Supplementary Fig. S3c). Conversely, sub 2/3 are observed in all tissues, which present a strengthened signature of lymphoid cell-mediated immune responses, such as cell killing, leukocyte-mediated cytotoxicity, natural killer cell-mediated cytotoxicity, interferon gamma (IFN-γ) signaling, etc (Supplementary Fig. S2a, b). The NK cytotoxicity-related genes (*GZMB, GZMH, LYZ, FGFBP2*), activating receptor genes (*KLRK, KLRC2, KLRC3*, *FCGR3A*), and maturation-related genes (*CX3CR1, ZEB2, PRDM1, KLF2*)^4,8^ are upregulated in these two sub-groups (Supplementary Fig. S3a, b). Consistently, the cytotoxicity score, an index of immune effector gene, of sub 3 was much higher than that of sub 1/2, suggesting stronger cytotoxicity ability of sub3 (Supplementary Fig. S3d).

The *t*-SNE plots presenting NKs and tumor cells show a discrete clustering of CTCs and tumor cells from solid lesions, implying discriminate molecular expressions and interactions (Supplementary Fig. S4a–c). Although the molecular interactive analysis shows a similar pattern of intensities among each group of NKs and tumor cells from different tissues (Supplementary Fig. S4d–f), the detailed dissection of immune-related molecular pairs unravel several distinct characters (Fig. 1f and Supplementary Fig. S4g–i). The interactive molecular pairs in primary and metastatic solid lesions present similar profiles. Remarkably, a unique interactive pattern of the immune checkpoint is observed between CTCs and NKs of sub 2/3). In addition to the previously reported molecular pairs (HLA-E and CD94-NKG2s)^3^, a group of immune checkpoints, including HLA-C:KIR2DL1 and HLA-C:KIR2DL3, are identified between CTCs and NKs (Fig. 1f). Similar to the engaging pattern of HLA-E:CD94-NKG2A, the enhancement of immune checkpoint HLA-C:KIR2DLs is mainly contributed by increasing level of HLA-C in CTCs (Fig. 1g). The levels of *KLRC1* (NKG2A), *KIR2DL1*, and *KIR2DL3* are relatively consistent among NKs from the hepatic portal vein (HPV) and solid lesions (Supplementary Fig. S4j–l). The high expressional levels of HLA-C and HLA-E are not limited to PDAC but also observed in multiple cancer types (Supplementary Fig. S5a, b). Importantly, both HLA-E and HLA-C are simultaneously upregulated in CTCs (Supplementary Fig. S5c–h). The potential mechanism underlying this upregulation is their similar promoter structure, which is regulated by platelet-derived RGS18 via the AKT-GSK3β-CREB1 axis^3,9^. Notably, the distribution of molecules KIR2DL1/3 and NKG2A shows a group-dependent pattern. The high levels of KIR2DL1/3 and NKG2A are observed in NK sub 3 and sub 2, respectively (Supplementary Fig. S5i–k). The results of multivariate kernel density estimation (a calculation based on the mRNA level with single-cell RNA-seq data) and flow cytometry analysis indicate that NKG2A^+^ and KIR2DL1^+^ NK populations are relatively exclusive (Fig. 1h–j and Supplementary Fig. S6a–c). The percentages of NKG2A^+^/KIR2DL1^+^ NKs measured by two independent methodologies are as low as 1.65% and 5.18% on average, respectively. Similar results were observed in NKG2A^+^ and KIR2DL3^+^ NK populations (Supplementary Fig. S5l). The results are consistent with previous reports that NKG2A and KIR2DL1/3 are mutually exclusively expressed on NKs^10,11^. Considering both NKG2A and KIR2DL1/3 serve as negative immune checkpoints on NKs, we then hypothesize that NKG2A^+^ and KIR2DL1/3^+^ NK populations surveil CTCs in blood circulation individually, like a complementary double prevention mechanism. CTCs escape this dual-surveillance by engaging HLA-E and HLA-C molecules simultaneously.

In the previous study, we efficiently prevented tumor metastasis by blockading the immune checkpoint HLA-E:CD94-NKG2A in a PDAC mouse model^3^. According to the results above (Fig. 1h–j), this treatment may only activate ~15.7%–54% of NKs. It also suggests that blocking HLA-C:KIR2DL1/3 may mobilize more NKs (additional ~12%–51.3%). Inspired by this observation, we wonder whether or not the elimination efficiency of CTCs mediated by NKs can be further improved by combining the disruption of immune checkpoints HLA-C:KIR2DL1/3 and HLA-E:CD94/NKG2A. Firstly, we performed the NK-killing assay in the presence of specific blockade antibodies against human NKG2A (Monalizumab) and KIR2DL1/3 (Lirilumab), finding that the combo blockade significantly promotes the tumor-killing activity of NKs and the secretion levels of IFN-γ (Supplementary Fig. S7a, b). To further validate the function of these immune checkpoints in vivo, we constructed mouse lung metastatic models that mimic extreme scenarios in clinics by injecting luciferase-labeled KPC cells (KPC-Luc) with or without the manipulation of H2-T23 (the mouse analog gene of HLA-E) and H2-D1 (the mouse analog gene of HLA-C) (Supplementary Fig. S7c–f). The tumor lung metastasis was dynamically monitored by optical in vivo imaging. Fifteen days after inoculation, the lungs were dissected, and the metastatic nodules were counted (Fig. 1k–n). The measurements of fluorescence intensity and metastatic nodule counting show that overexpression of H2-D1 significantly promoted lung metastasis. Fluorescence intensity measurement indicates that compared with control group, single disruption of immune checkpoints by knocking down either H2-T23 or H2-D1 remarkably reduces the metastasis by 80.5% and 80.1%, respectively. Similar reducing rates are obtained by counting the metastatic nodules (76.7% and 77.3%, respectively) (Supplementary Fig. S7g, h). Combinatorial knockdown of H2-T23 and H2-D1 further improves the metastatic reducing rates to 92.2% and 89.6%, evaluated by fluorescence intensity and nodule count, respectively. These observations are confirmed by Hematoxylin and Eosin (H&E) staining (Fig. 1o). The results were further validated by another independent cohort (Supplementary Fig. S7i, j). The Kaplan–Meier curve shows that simultaneously knocking down of H2-D1 and H2-T23 significantly promotes the overall survival of lung metastatic mice after tumor inoculation (Fig. 1p). In summary, NKs immune surveil the tumor cells (CTCs) during the hematogenous metastasis process. To escape this surveillance, CTCs engage negative immune checkpoint molecules, HLA-E and HLA-C, to suppress the NK function by binding to counterpart molecules NKG2A and KIR2DL1/3. Compared with single-blockade of these two immune checkpoints, dual-blockade of them may potentially achieve a better efficacy in terms of metastasis prevention by increasing the proportion of activated NKs and the chance of CTCs–NKs interaction (Fig. 1q).

Tumor-distant metastasis via the bloodstream is a major contributor to patient mortality^12^. Preventing metastasis by eliminating tumor cells in blood circulation is one of the most promising strategies theoretically. Studies have shown that neutrophils and Treg cells in the blood circulation can facilitate the survival and immune escape of CTCs^13,14^. Conversely, NKs are regarded as the most critical killer cells in CTCs immune surveillance^15^, and efforts should be made to mobilize NKs. Thus, identifying both negative and positive immune checkpoints between NKs and CTCs is important for preventing tumor metastasis. Here, we identified the exclusive negative immune checkpoints between CTCs and NKs, namely HLA-C:KIR2DL1/3 and HLA-E:CD94/NKG2A. Blocking these checkpoints simultaneously has been shown to significantly inhibit tumor metastasis. As monalizumab, the antibody against NKG2A, and lirilumab, a pan anti-KIR antibody, have already undergone clinical trials, the combination of these two treatments has the potential to control tumor metastasis in PDAC patients. Moreover, as both NKG2A^+^ and KIR2DL1/3^+^ NKs are ineffective against CTCs, the proportion of these cells may serve as an indicator for the effectiveness of NKs-based cancer therapy and the prognosis of PDAC. Additionally, we also identified other immune checkpoints of NKs in our system, such as KLRG1, KLRD1, KLRK1, KLRC2, and KLRC3, which may also get involved in the NKs-mediated immune surveillance (Supplementary Figs. S3a and S4d–f). Nevertheless, further validation is needed to determine the specific functions of these immune checkpoints in the elimination of CTCs.

### Supplementary information

Supplementary Information

Ethic Approval

## Data Availability

The raw sequencing data that support the findings of this study are deposited at National Genomics Data Center (NGDC, https://ngdc.cncb.ac.cn/) under GSA-human: HRA003672 (https://ngdc.cncb.ac.cn/gsa-human). The processed data of scRNA have been deposited at NGDC under OMIX: OMIX002487 (https://ngdc.cncb.ac.cn/omix/releaseList/). Any additional information required to reanalyze the data reported in this paper is available from the corresponding author upon request.
