# Supplementary material for: Immune checkpoints HLA-E:CD94-NKG2A and HLA-C:KIR2DL1 complementarily shield circulating tumor cells from NK-mediated immune surveillance
Source: Cell Discov. 2024 Feb 9;10:16. doi: 10.1038/s41421-024-00646-3 (PMC10858264; doi:10.1038/s41421-024-00646-3)
Supplement: Supplementary file 1 — Supplementary Information [file 41421_2024_646_MOESM1_ESM.pdf]

Supplementary Information

Materials and Methods

Patients and specimens

All human studies conducted in this study were approved by the Ethics Committee on Biomedical Research of West China Hospital. Before collecting the clinical biopsies, informed consent was obtained from the patients. The primary tumor tissues, patient-matched liver metastatic tumor lesions, and HPV blood were acquired from patients with liver metastatic PDAC for scRNA-seq, which have been previously described[1]. In addition, blood samples from PDAC patients were collected for flow cytometry analysis to detect the expression levels of NKG2A and KIR2DL1 on NK cells.

Cells and mice

Mouse PDAC cell lines KPC was obtained from the Department of Pancreatic Surgery at West China Hospital (Chengdu, China). HEK-293T cell line was purchased from the National Collection of Authenticated Cell Cultures (Shanghai, China). The PDAC cell lines SU86.86 were purchased from the American Type Culture Collection (ATCC). The KPC, SU86.86, and HEK-293T cells were cultured in RPMI-1640 and DMEM medium with 10% fetal bovine serum (FBS, Gibco), respectively. All cells were cultured aseptically at 37°C in humidified incubators with 5% CO_2.

Six-week-old female Balb/c nude mice were purchased from Beijing Vital River Laboratory Animal Technology Co., Ltd (Beijing, China). The animals were housed and maintained under SPF condition in facilities and treated humanely throughout the studies. All animal experiments were performed according to the protocols approved

by the Ethics Review Committee of Animal Experimentation of Sichuan University.

Single-cell and bulk RNA-Seq datasets collection

The single-cell transcriptomes of PDAC primary tumors, CTC circulations, and metastatic lesions were obtained from our previous scRNA-seq data (OMIX: OMIX002487)[1]. To highlight the prevalent phenomenon of elevated HLA-E and HLA-C expression in CTCs, we curated a compendium of previously published CTC sequencing datasets, encompassing both single-cell and bulk RNA-seq data. Specifically, we incorporated scRNA-seq data from hepatocellular carcinoma (HCC; accession: CNP0000095)[2]. Additionally, we compiled bulk RNA-seq data across diverse cancer types, including HCC (accession: GSE117623)[3], pancreatic ductal adenocarcinoma (PDAC; accession: GSE144561)[4], breast cancer (BRCA; accessions: GSE67939, GSE86978)[5, 6], colon adenocarcinoma (COAD; accession: GSE74369)[7], and melanoma (SKCM; accession: GSE38495)[8].

Process of scRNA PDAC data

For the processing of scRNA-seq data on CTCs and paired PDAC tumor cells, we employed the widely used cellranger software (version 3.0.0) provided by 10× Genomics. This software suite is tailored for the analysis of single-cell transcriptomic data generated through the Chromium platform. Initially, the raw sequencing data was aligned to the GRCh38 genome reference using cellranger's alignment module. Subsequently, the cellranger count pipeline was employed to generate feature-barcode matrices, which serve as the foundation for downstream analytical steps, including clustering and gene expression analysis. To ensure data quality, a stringent quality

control protocol was implemented. This involved filtering out cells exhibiting either an excessive (>7,500) or minimal (<200) number of expressed genes, as these extremes are indicative of low-quality cells or potential doublets with aberrant gene counts. Cells with mitochondrial gene expression levels surpassing 25% were also excluded, as heightened mitochondrial content often signifies cell stress or damage. Furthermore, genes expressed in fewer than three cells within a sample were removed. The identification of doublets was carried out by detecting cells expressing multiple cell markers. After the application of these quality control measures, a total of 74,206 single cells from 18 samples were retained, encompassing 26,808 distinct features for subsequent comprehensive downstream analysis.

Dimensionality reduction and clustering

To unravel the complexity within the single-cell transcriptomic data, a series of dimensionality reduction and clustering steps were executed employing the Seurat R package (version 4.0.1)[9]. After quality control of cells and genes, the "LogNormalize" normalization technique was applied, ensuring that each cell's gene expression was adjusted based on the total expression while incorporating a scaling factor of 10,000 and log-transformation. The identification of highly variable genes was achieved through the FindVariableFeatures function, followed by data normalization using the ScaleData function to transform gene expression values into z-scores. Principal component analysis (PCA) was then performed on the scaled data, focusing on 2,000 highly variable genes. An optimal number of 50 principal components, determined through an Elbow plot analysis, was selected for subsequent analyses. Graph-based

clustering, an essential facet of revealing cellular heterogeneity, was executed using the FindNeighbors function to establish a K-nearest neighbor (KNN) graph, refining edge weights between cells. Subsequently, the Louvain algorithm was employed through the FindClusters function, iteratively grouping cells based on the selected 50 principal components and guided by a resolution parameter set at 1. For visualization of the single-cell transcriptomic landscape in a reduced dimension, t-SNE was implemented. The t-SNE algorithm employed 50 principal components as input to project the data into a lower-dimensional space, facilitating the visualization of distinct cell clusters. This integrated approach allowed us to effectively identify and differentiate diverse cell populations within the complex single-cell transcriptomic dataset, providing insights into the underlying cellular heterogeneity and functional diversity.

Differential expression analysis

To identify genes that contribute to the uniqueness of different clusters, the FindMarkers function was used. This function employs Wilcoxon rank sum test to assess differential expression between clusters. Genes demonstrating statistically significant differential expression were ascertained by considering adjusted p-values and fold changes, thereby highlighting genes that are pivotal in distinguishing cellular identities. The min.pct argument was set as 0.25, and the logfc threshold argument was set as 0.25.

Cell annotation

Accurate cell annotation and sub-typing were achieved through a multi-step approach encompassing the sciBet R package (version 1.0)[10], conical marker genes, and

inferCNV outcomes[11]. Initially, cell annotations were established based on distinctive marker expressions and inferCNV results. Epithelial cells were identified by their co-expression of *EPCAM*, *KRT8*, and *KRT18*. Fibroblasts were recognized through *FAP* and *COL1A1* expressions. Endothelial cells were pinpointed by *VWF* and *PECAM1* co-expression. Immunocytes were annotated by their expression of *PTPRC*. Additionally, CTCs within blood tissue were discerned via the *PTPRC*$^-$*CD9*$^+$*PPBP*$^+$ signature and inferCNV-derived information. Subsequently, immunocytes are categorized into 8 lymphoid subtypes, and 7 myeloid subtypes.

Copy number variation detection from sc-RNAseq data

Detection of copy number events within sc-RNAseq data was facilitated by utilizing the CopyKAT R package (version 1.0.5), enabling the generation of copy number variation (CNV) profiles. CopyKAT employs advanced integrative Bayesian methods designed to identify genome-wide aneuploidy patterns at a resolution of 5 megabases (MB). Cells demonstrating substantial genome-wide aneuploidy were earmarked as putative tumor cells. The analysis employed the UMI count matrix as input, while retaining default parameters for other settings.

Enrichment analysis and differential gene set analysis

To uncover enriched biological pathways and functions within the single-cell transcriptomic data, we used gene sets encompassing C2.CP.KEGG, C2.CP.REACTOME, and C5.BP categories. These gene sets were sourced from the Molecular Signatures Database (MSigDB v.7.1)[12]. We executed SingleCellSignatureExplorer (v.3.6) to calculate single-cell signature scores[12],

providing a quantitative measure of pathway activation status. For subsequent differential expression analysis of these signature scores, we leveraged the limma R package (v.3.42.0)[13]. Enriched gene sets were ascertained based on an adjusted p-value threshold of < 0.05.

Exploring cell-cell interactions via ligand-receptor analysis

Exploring the intricate network of cell-cell communication within the single-cell transcriptomic milieu was undertaken using CellPhoneDB (version 2.0)[14]. We delved into the landscape of ligand-receptor interactions between tumor cells and immunocytes, utilizing single-cell RNA-seq data. The reference repository of CellPhoneDB was sourced from diverse databases including UniProt, Ensembl, PDB, the IMEx consortium, and IUPHAR, yielding an extensive compilation of 1,396 receptor-ligand interactions. To dissect the interactive dynamics, potential molecules governing these interactions were extracted from the scRNA-seq data and compared against the reference repository. The characterization of interactions within each cell type hinged on two parameters: the mean value derived from the combined expression of ligands and receptors and the p-value obtained through an empirical shuffling algorithm. The focus on significant interactions, particularly those associated with immune checkpoints (determined by $p < 0.05$ and a mean (Molecule 1, Molecule 2) > 0.5), unveiled the complex choreography of immune modulation. In the network plot, the interaction weight score represented the cumulative gene expression counts of significant ligand-receptor pairs between distinct cell types. This comprehensive

analysis illuminated the intricate communication networks shaping immune responses and cell fate determinants within the context of single-cell transcriptomic heterogeneity.

Public CTCs RNA-seq data analysis

For HCC CTC single-cell sequencing data, we performed data filtering, clustering, and dimensionality reduction analysis as described before. Notably, parameter settings of PCs and resolution were fixed at 10 and 0.2, respectively. For bulk RNA-seq data, the six bulk RNA-seq datasets were merged firstly. Then, following data filtering, clustering, and dimensional reduction analysis, the PCs and resolution parameter were fixed at 10 and 0.2. This comprehensive approach enabled the comparison of HLA-E and HLA-C expression in CTCs across distinct datasets.

Flow cytometry analysis

To analyze the expression of NKG2A$^+$ NKs and KIR2DL1$^+$ NKs in blood, PBMC from PDAC patients were collected and stained with indicated antibodies for flow cytometry analysis. Briefly, the PBMC were isolated from PDAC patients' blood by human lymphocyte separation medium (DAKEWEI, #7111012) and washed with HBSS. The collected cells were then resuspended in cell stain buffer (BioLegend, #420201) and incubated with Fc Receptor Blocking Solution (Biolegend, #422302) for 15 minutes to avoid nonspecific binding. Subsequently, cells were incubated with antibodies against CD3 (BioLegend, #300306), CD16(BioLegend, #302040), CD56 (BioLegend, #362554), NKG2A (BioLegend, #375103), and KIR2DL1 (BioLegend, #339510) for 30 minutes on ice. Then cells were washed and resuspended in cell stain buffer for flow cytometry analysis (BD Biosciences, SanJose, CA). The data were

processed by Flojo_V10 software. The $CD3^-$ $CD16^+$ $CD56^+$ NKs were used to analyze the expression of NKG2A and KIR2DL1 on NKs.

Cell transfections

Mouse H2-D1 genes were synthesized and subcloned into lentivirus vector pRRLsin.cPPT.CMV.Blasticidin (denoted as CPPT). shRNA (knockdown) sequence specifically targeted to H2-T23, and H2-D1 were synthesized and subcloned into PLKO vector to generate lentiviruses. Recombinant lentiviruses were generated by third-generation lentiviral packaging using human embryonic kidney (HEK) 293T cells. HEK293T cells were transfected using calcium phosphate. Then the lentiviruses were harvested and transfected with tumor cells. After 24 hours, the tumor cells were screened with blasticidin (for CPPT vector, Invivogen, #ant-bl-1) or puromycin (for PLKO vector, Selleck, #S7417) to obtain highly transduced cells. shRNA primers used to construct the vector are listed in Table S1.

Cytotoxicity assay

As our previously described[1], the cytotoxicity of NKs to tumor cells were detected by LDH-Glo™ Cytotoxicity Detection Kit (Promega, #J2381). In brief, SU86.86 cells were seeded in 96-well plates at a density of 2.5×10^3 cells/well and incubated overnight. On the next day, NKs were isolated from the blood of PDAC patients and pre-incubated with 200 μg/ml Monalizumab (BioChemPartner, Cat#BCP46059) and 200 μg/ml Lirilumab (MedChemExpress, HY-P99208). Then, NKs were dispensed into the wells of target cells at 10:1 effector:target (E: T) ratios in triplicate wells. After 24 hours, the samples were collected and detected with LDH Detection Reagent (Promega,

Cat#J2381) and IFN-γ ELISA kit (Dakewe, Shenzhen, China, #1110002). The percentage of tumor cell lysis was defined as follows: Cytotoxicity (%) = 100 × (Experimental LDH Release – Medium Background) / (Maximum LDH Release Control – Medium Background).

Quantitative reverse transcription PCR

The procedures of RNA extraction and quantitative reverse transcription PCR (qPCR) were described in our previous study[15]. Briefly, after the KPC cells were transfected with corresponding lentiviruses for 48 hours, the cells were lysed with Trizol (Life Technologies, #15596-026) for RNA extraction. The extracted RNA was then used to synthesize cDNA with the RevertAid First Strand cDNA synthesis Kit (Thermo, #K1622). Quantitative PCR was performed using Universal SYBR® Green Supermix (Bio-Rad, #172-5121). GAPDH was used as the reference gene for relative quantification. Primers specifically targeted H2-T23 and H2-D1 were designed to detect the expression of H2-T23 and H2-D1 in mouse KPC cells. Primers used for qPCR are listed in Table S2.

***In vivo* tumor metastasis study**

To illustrate the role of immune checkpoint molecules H2-T23 (HLA-E) and H2-D1 (HLA-C) on CTCs-mediated tumor metastasis, Balb/c nude mice were intravenously inoculated with luciferase-tagged KPC cells (KPC-Luc, 5×10^4 cells) that were pre-overexpressed H2-D1 or pre-knocked down H2-T23, H2-D1, and H2-T23 + H2-D1. After 15 days, D-luciferin potassium was injected into the mice and they were analyzed using the IVIS Spectrum Imaging System. Following imaging, the mice

were sacrificed and their lung tissues were collected for pathological examination.

In another independent lung metastasis model, we investigate the effects of H2-T23 and H2-D1 on mice survival. As described above, Balb/c nude mice were given KPC-Luc cells (1.2×10^5 cells/mice) that had been pre-overexpressed with H2-D1, or pre-knocked down with H2-T23, H2-D1, and H2-T23 + H2-D1. The mice's survival was monitored for 35 days and the Kaplan-Meier survival curves were plotted.

QUANTIFICATION AND STATISTICAL ANALYSIS

The R software (v4.0) was utilized for single-cell RNA-seq data analysis. To plot the Kaplan–Meier survival curves, the survival and survminer R packages were employed. Flow cytometry data was analyzed using FlowJo_V10. To test the significance of DEG analysis, the Wilcoxon rank-sum test was utilized, while the two-sided Wilcoxon test was applied to test the significance of gene expression. For comparison between two groups, the independent-samples t-test was employed. Statistical significance thresholds were set at * P value < 0.05; ** P value < 0.01; *** P value < 0.001, **** P value < 0.0001.

Supplementary Figures

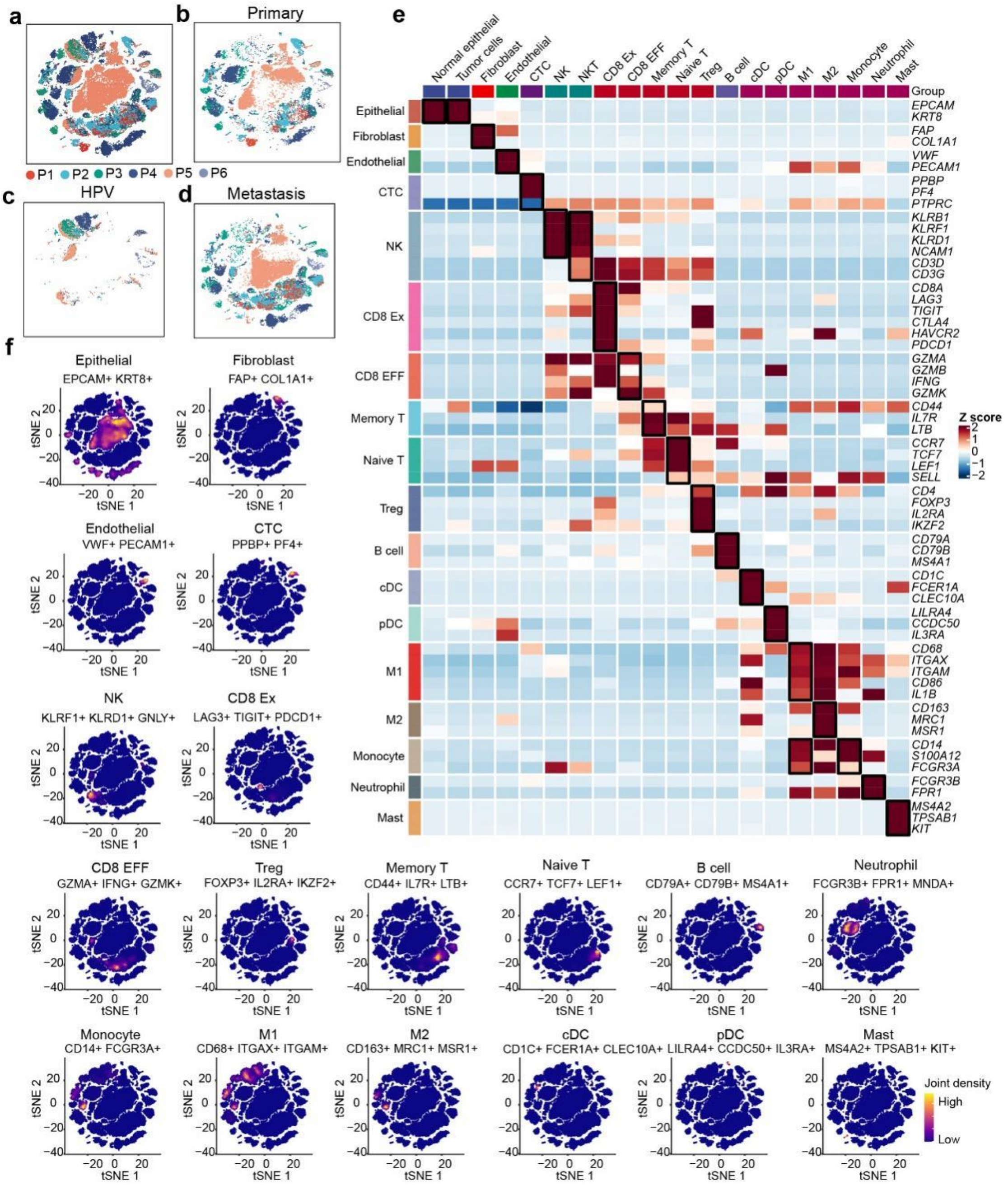

Figure S1. Single cell RNA-seq analysis of the transcriptomic landscape of PDAC primary tumors, metastatic lesions, and CTCs. a-d The t-SNE plots show the patient (**a**) and tissue (**b-d**) origin of sequenced cells. **e** Heatmap shows the expression of marker genes in each cell subtype. Total 19 kinds of cell subtypes were identified, including 29,930 epithelial (*EPCAM*$^{+}$, *KRT8*$^{+}$) cells, 1,675 fibroblasts (*FAP*$^{+}$,

COLA1^{+}), 876 endothelial (*VWF*$^{+}$, *PECAM1*$^{+}$) cells, 523 CTCs (*PTPRC*$^{-}$, *PPBP*$^{+-}$, *PF4*$^{+}$), NK cells (*KLRD1*$^{+}$, *KLRF1*$^{+}$), NK-T cells (*CD3D*$^{+}$, *CD3E*$^{+}$, *KLRD1*$^{+}$, *KLRF1*$^{+}$), CD8 exhausted T-cells (CD8 Ex; *CD8A*$^{+}$, *PDCD1*$^{+}$, *LAG3*$^{+}$, *TIGIT*$^{+}$, *CTLA4*$^{+}$), CD8 effector T-cells (CD8 EFF; *CD8A*$^{+}$, *IFNG*$^{+}$, *GZMA*$^{+}$), memory T-cells (*CD3D*$^{+}$, *CD3E*$^{+}$, *CD44*$^{+}$, *IL7R*$^{+}$, *LTB*$^{+}$), Naïve T-cells (*CD3D*$^{+}$, *CD3E*$^{+}$, *CCR7*$^{+}$, *TCF7*$^{+}$, *SELL*$^{+}$, *LEF1*$^{+}$), Treg (*FOXP3*$^{+}$, *IL2RA*$^{+}$), B cells (*CD79A*$^{+}$, *CD79B*$^{+}$), classical DCs (*CD1C*$^{+}$, *FCER1A*$^{+}$, *CLEC10A*$^{+}$), plasma DCs (pDC; *LILRA4*$^{+}$, *IL3RA*$^{+}$), M1 macrophages (*FCGR3A*$^{+}$, *CD68*$^{+}$, *ITGAX*$^{+}$, *ITGAM*$^{+}$), M2 macrophages (*CD163*$^{+}$, *MRC1*$^{+}$, *MSR1*$^{+}$), monocyte (*CD14*$^{+}$, *S100A12*$^{+}$, *FCGR3A*$^{+}$), neutrophil (*CD14*$^{-}$, *FCGR3B*$^{+}$, *FPR1*$^{+}$), and mast cells (*MS4A2*$^{+}$, *TPSAB1*$^{+}$, *KIT*$^{+}$). **f** The expression of the marker genes for the indicated cell subtypes is presented on the t-SNE plots. Colors represent the logarithm of normalized data, and each dot represents a single cell.

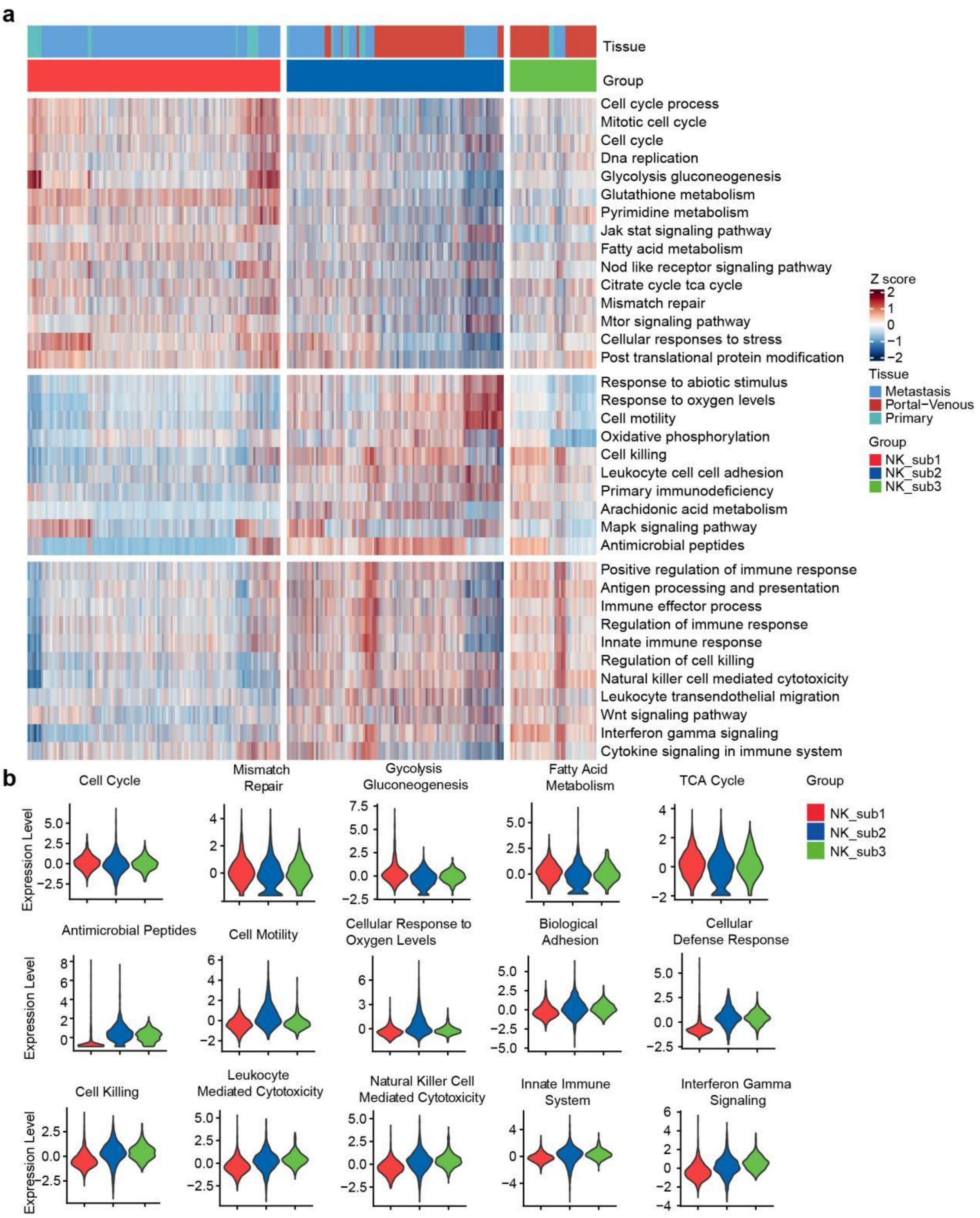

Figure S2. The functional enrichment analysis in NK sub 1, 2, and 3. **a** Heatmap presents the differentially enriched gene sets between NK sub 1, 2, and 3.The rows represent gene sets, and columns represent cells annotated with cell type and tissue origin. Colors present GSVA Z scores for each cell. **b** The representative signature pathways in NK sub 1, 2, and 3.

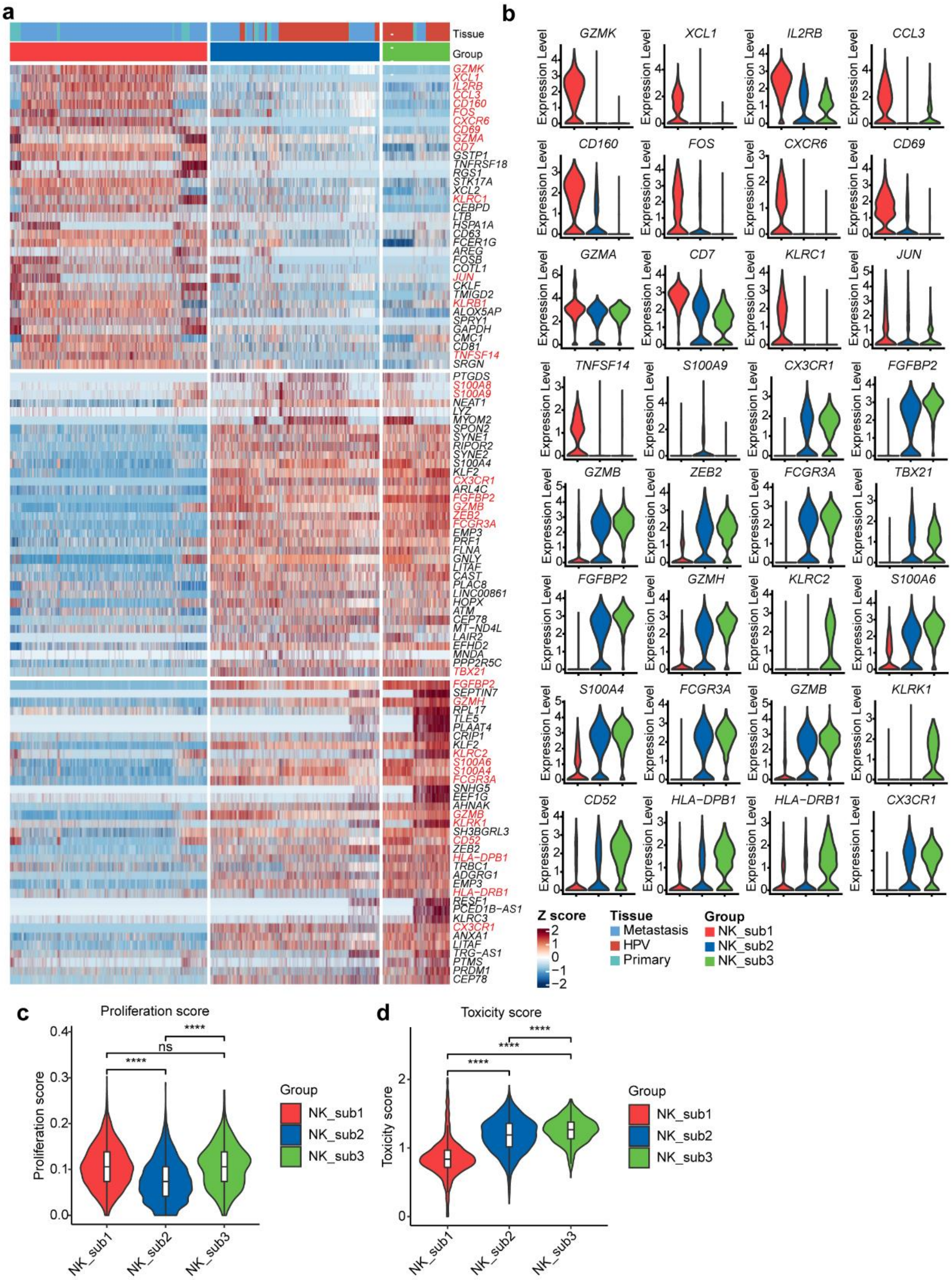

Figure S3. Characterizing the transcriptional signature of NKs. a The top50 DEGs between NK sub 1, 2, and 3 are displayed based on the normalized expression. Genes related to cell cycle (navy blue), metabolism (green), cytokines (brown),

cytotoxicity (red), NK activating receptor (yellow), proinflammatory factor (purple), NK maturation (pink) were highlighted, respectively. **b** The boxplots show the normalized expression levels of cell cycle (*FOS, FOSB, JUN, CEBPD*), glycometabolism and lipid metabolism (*CD160, GSTP1, AREG, ALOX5AP*), cytokines (*GZMK, XCL1, XCL2, CCL3*), cytotoxicity (*GZMB, GZMG, LYZ, FGFBP2*), NK activating receptor (*KLRK1, KLRC2, KLRC3, FCGR3A*), proinflammatory factor (*S100A8, S100A9, S100A6, S100A4*), and NK maturation-related genes (*CX3CR1, ZEB2, PRDM1, KLF2*) in NK sub 1, 2, and 3. **c, d** The proliferation and cytotoxicity score of NK sub 1, 2, and 3 were calculated based on the proliferation (**c**) and cytotoxicity (**d**) related genes. Two sides Wilcoxon test, **** P value < 0.0001.

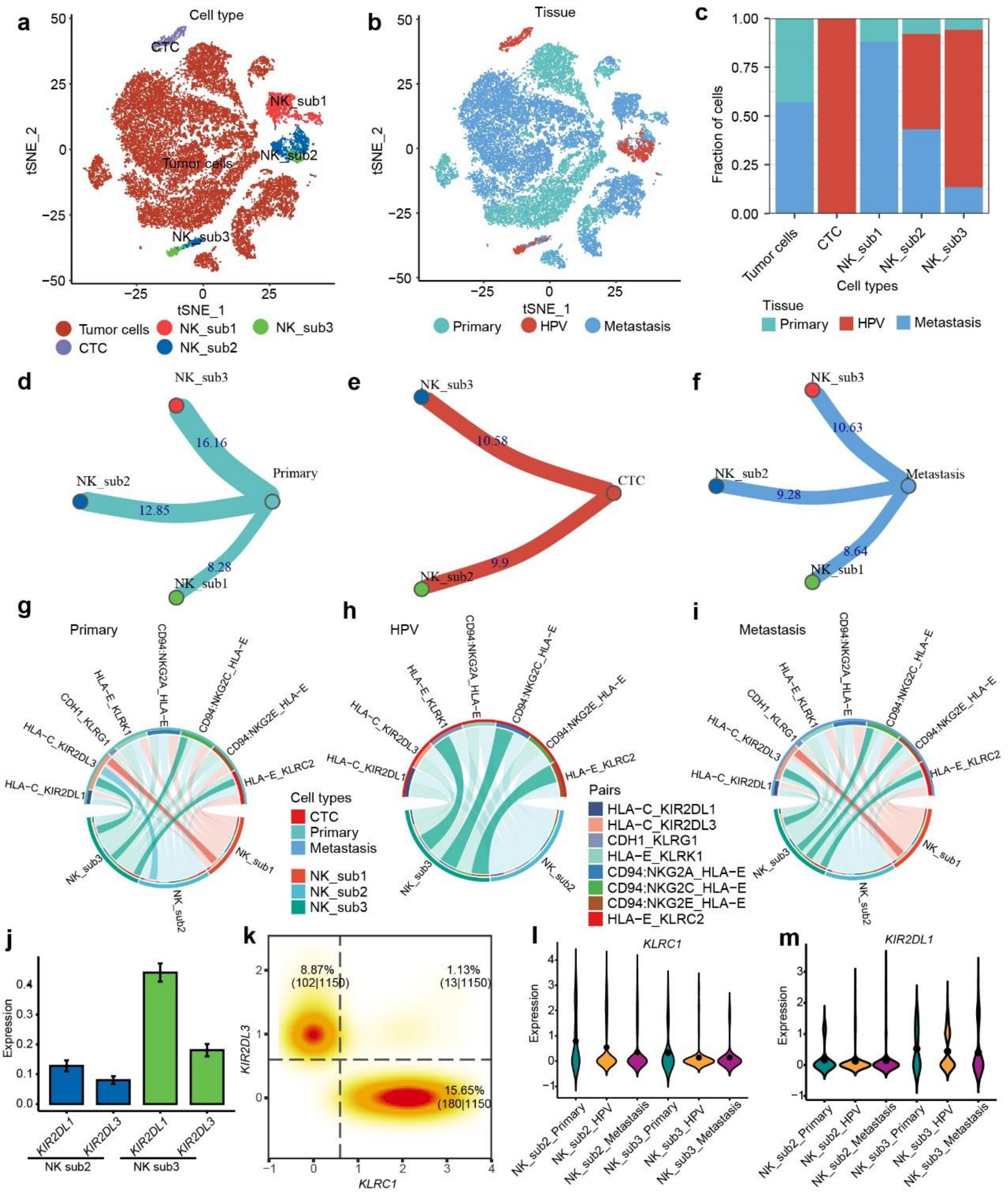

Figure S4. The interaction analysis between NKs and CTCs/tumor cells in blood, primary, and metastatic lesions. a, b The t-SNE plots present the tumor cells and NKs with cell types (**a**) and tissue (**b**) origin. The color codes are displayed at the bottom of each panel. **c** Bar plot shows the tissue proportion of each cell type. Colors represent cell origin. **d-f** The potential ligands and receptors interaction between tumor cells/CTCs and NKs in the primary tumor (**d**), blood circulation (**e**), and metastasis

tumor (**f**). The thickness of the line represents the number and expression level of ligand-receptor pairs. **g-i** CellPhoneDB analysis of identified immune checkpoint between tumor cells/CTCs and NKs in the primary tumor (**g**), blood circulation (**h**), and metastasis lesions (**i**). **j-l** Violin plots show the expression of *KLRC1* (**j**), *KIR2DL1* (**k**) and *KIR2DL3* (**l**) on NK cells from primary tumors, HPV blood, and metastatic lesions. The black dots present the mean expression levels of each group.

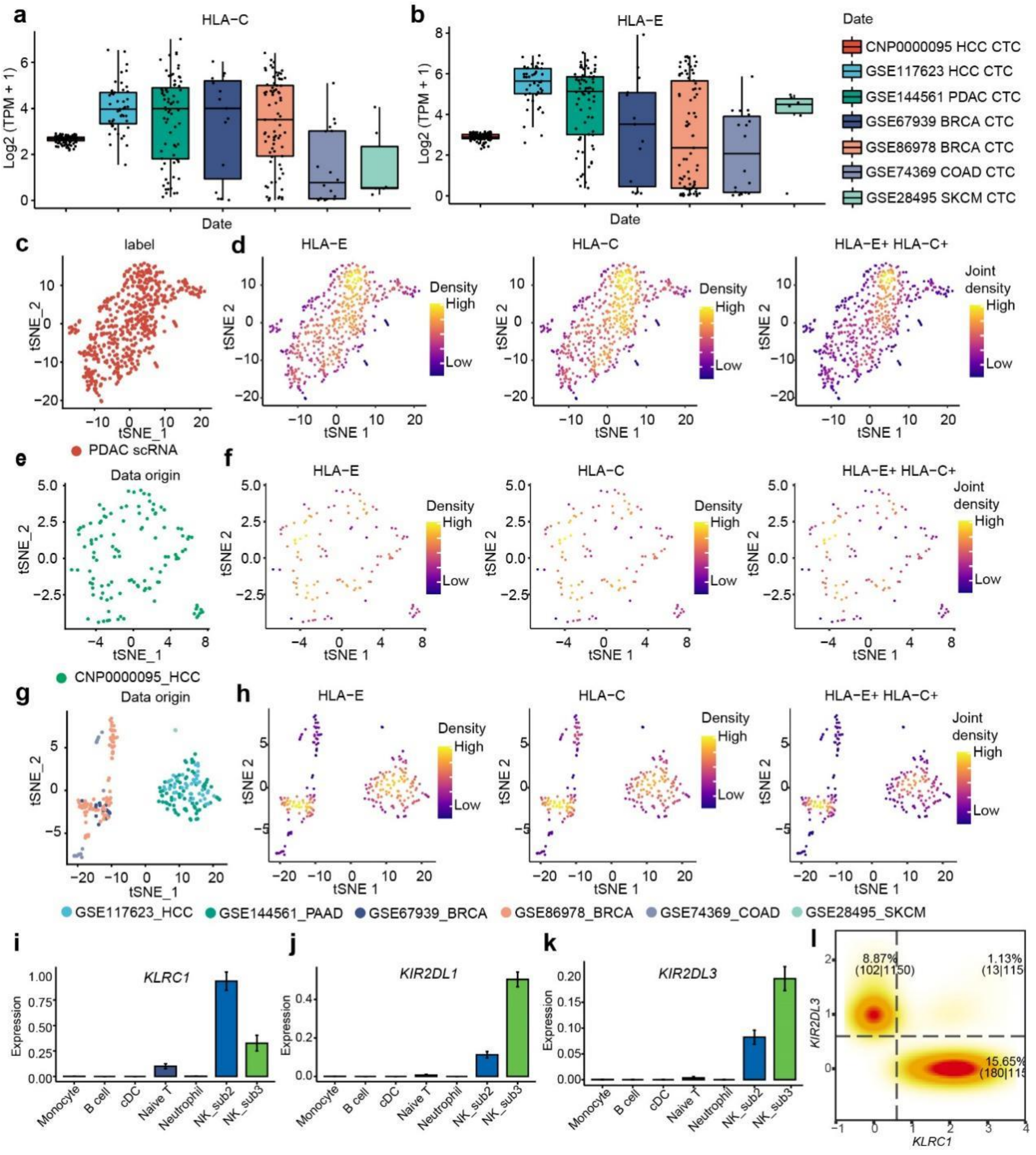

Figure S5. Validation of the expression of HLA-E and HLA-C in multiple cancer-derived CTCs. a, b Box plots present the expression of *HLA-E* (**a**) and HLA-C (**b**) on CTCs from multiple solid cancers. The scRNA-seq data of CTC were obtained from public databases of human-derived CTCs, including hepatocellular carcinoma (HCC; CNP0000095, GSE117623), PDAC (GSE144561), breast cancer (BRCA; GSE67939, GSE86978), colon adenocarcinoma (COAD; GSE74369), and melanoma (SKCM; GSE38495). **c, d** The t-SNE plots present the expression levels of *HLA-E* and *HLA-C* on each CTCs from our dataset. Colors represent the logarithm of normalized data, and each dot represents a single cell. **e, f** The expression of *HLA-E* and *HLA-C* of CTCs from the HCC scRNA-seq dataset (CNP0000095) are projected on the t-SNE plots. **g, h** The bulk-RNA-seq transcriptome data of CTCs from multiple malignancies are projected with t-SNE plot and the expression of *HLA-E* and *HLA-C* are displayed. **i-k** Bar plots show the relative expression levels of *KLRC1* (**i**), *KIR2DL1* (**j**) and *KIR2DL3* (**k**) in immunocytes from HPV blood. Mean ± SE. **l** Multivariate kernel density plots depict the proportion of *KLRC1* (NKG2A) and *KIR2DL3* on HPC-derived NKs.

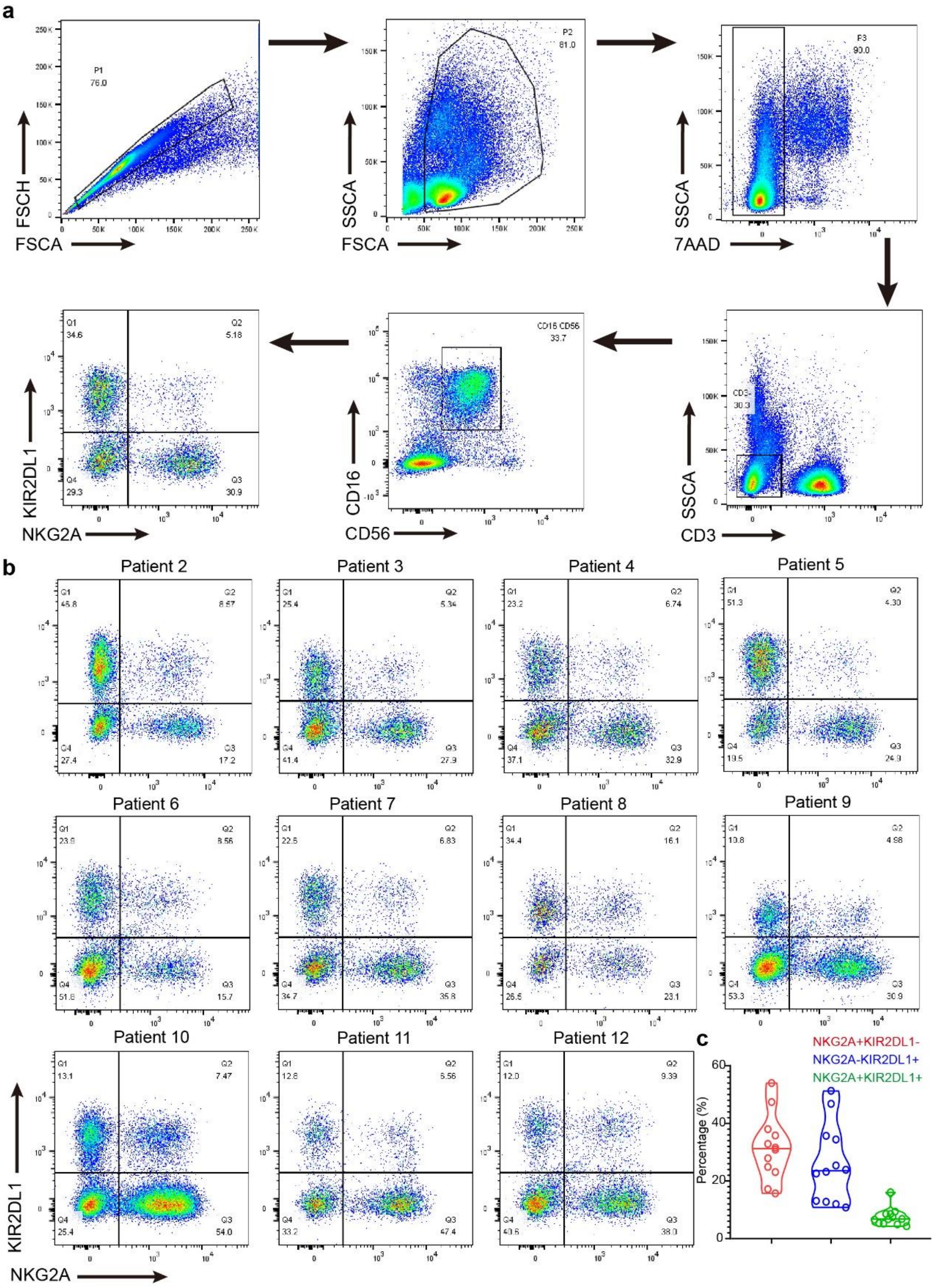

Figure S6. Flow cytometry plots analyzed the expression of NKG2A and KIR2DL1 on the NKs of PDAC patient blood samples. a Gating strategy for the identification of NKs subtype populations. Starting from the upper left, arrows indicate direction of sub-gates. **b, c** Flow cytometry plots displayed (**b**) and quantified (**c**) the percentage of

NKG2A$^+$ and KIR2DL1$^+$ NKs in the blood from PDAC patients.

.

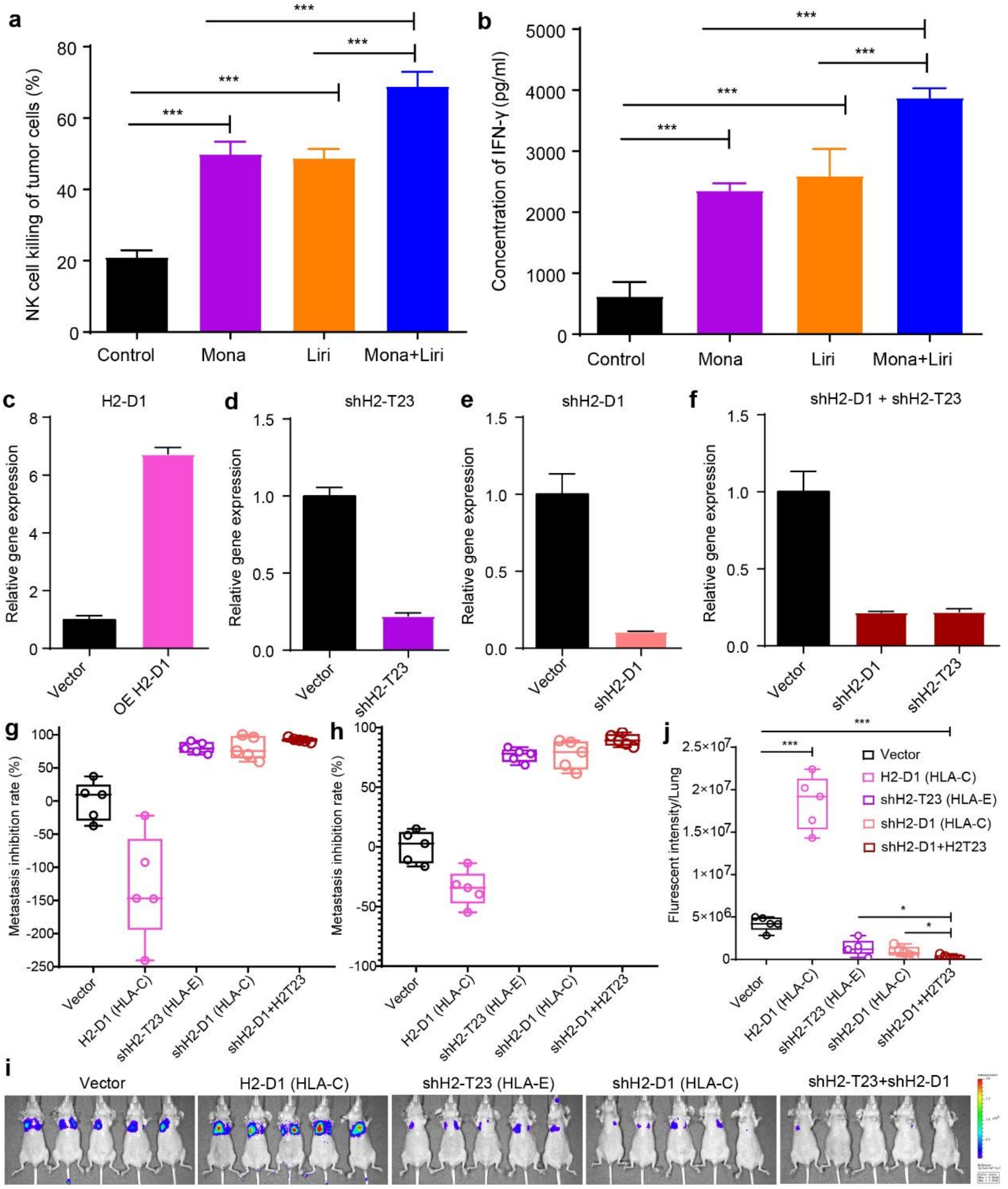

Figure S7. Interrupted the immune checkpoints HLA-E:CD94-NKG2A and HLA-C:KIR2DL1 efficiently prevents tumor metastasis. a The NKG2A and KIR2DL1/3 on NKs were blocked with Monalizumab and Lirilumab antibodies, respectively. Then, the cytotoxicity of NKs to PDAC SU86.86 cells was evaluated by LDH release assay.

The results were presented as mean ± SD, n = 3, *** $P < 0.01$, based on t-test. **b** After incubation with SU86.86 cells, the cytotoxic effector cytokines IFN-γ were detected by ELISA. The results were presented as mean ± SD, n = 3, *** $P < 0.01$, based on t-test. **c-f** The expression levels of H2-T23 (mouse homolog of HLA-E) and H2-D1 (mouse homolog of HLA-C) were detected in KPC cells with overexpression of H2-D1 (**c**), shH2-T23 (**d**), shH2-D1 (**e**), or H2-T23 + H2-D1 knockdown (**f**). **g, h** The metastasis inhibition rates were calculated according to the bioluminescence intensity (**g**) and tumor lung nodules (**h**) of Fig1. k-n. **i, j** During plotting the Kaplan–Meier survival curve, the bioluminescence signal intensities of metastasis tumors in the lungs were visualized (**i**) and quantified (**j**).

Table S1 Primer sequences in shRNA plasmid construction.

**Gene name**	**Sequences(5'-3')**
H2-D1 shRNA-1	CCCTCAGTTCTCTTTAGTCAA
H2-D1 shRNA-2	GGTACATCTCTGTCGGCTATG
H2-D1 shRNA-3	AGTGGTGCTGCAGAGCATTAC
H2-T23 shRNA-1	TCCTGGACCGCGAATGACATA
H2-T23 shRNA-2	ACATAGCCTCACAGATCTCTA
H2-T23 shRNA-3	AGATCTCTAAGCACAAGTCAG

Note: After evaluating the knockdown efficiency of these shRNA sequences, the H2-D1 shRNA-1 and H2-T23 shRNA-2 were utilized to develop stable cell lines with knocked-down expression of H2-D1 and H2-T23.

Table S2 Primer sequences for qPCR

**Gene name**	**Sequences (5'-3')**
GAPDH-F	AATGGGAAGCTTGTCATCAA
GAPDH-R	GTGAAGACACCAGTAGACTC
H2D1-F	TCTCACACACTCCAGCAGAT
H2D1-R	CCTCCAGGTAGGCCTTGTAA
H2T23-F	AGCACAAGTCAGAGGCAGTC
H2T23-R	ATGTGCCTTTGGAGGGTCTG

Reference

1 Liu X *et al. Cancer Cell*; **41**:272-287.e279 (2023).
2 Sun YF *et al. Nat Commun*; **12**:4091 (2021).
3 Bhan I *et al. Gastroenterology*; **155**:2016-2018.e2011 (2018).
4 Franses JW *et al. Nat Commun*; **11**:3303 (2020).
5 Aceto N *et al. Mol Cancer Res*; **16**:720-727 (2018).
6 Sarioglu AF *et al. Nat Methods*; **12**:685-691 (2015).
7 Cima I *et al. Sci Transl Med*; **8**:345ra389 (2016).
8 Ramsköld D *et al. Nat Biotechnol*; **30**:777-782 (2012).

9 Hao Y *et al. Cell*; **184**:3573-3587.e3529 (2021).
10 Li C *et al. Nat Commun*; **11**:1818 (2020).
11 Gao R *et al. Nat Biotechnol*; **39**:599-608 (2021).
12 Liberzon A *et al. Bioinformatics*; **27**:1739-1740 (2011).
13 Ritchie ME *et al. Nucleic Acids Res*; **43**:e47 (2015).
14 Efremova M *et al. Nat Protoc*; **15**:1484-1506 (2020).
15 Wang X *et al. Cell Rep Med*; **3**:100741 (2022).
