# Supplementary figures and images for: Immune checkpoints HLA-E:CD94-NKG2A and HLA-C:KIR2DL1 complementarily shield circulating tumor cells from NK-mediated immune surveillance
Source: Cell Discov. 2024 Feb 9;10:16. doi: 10.1038/s41421-024-00646-3 (PMC10858264; doi:10.1038/s41421-024-00646-3)

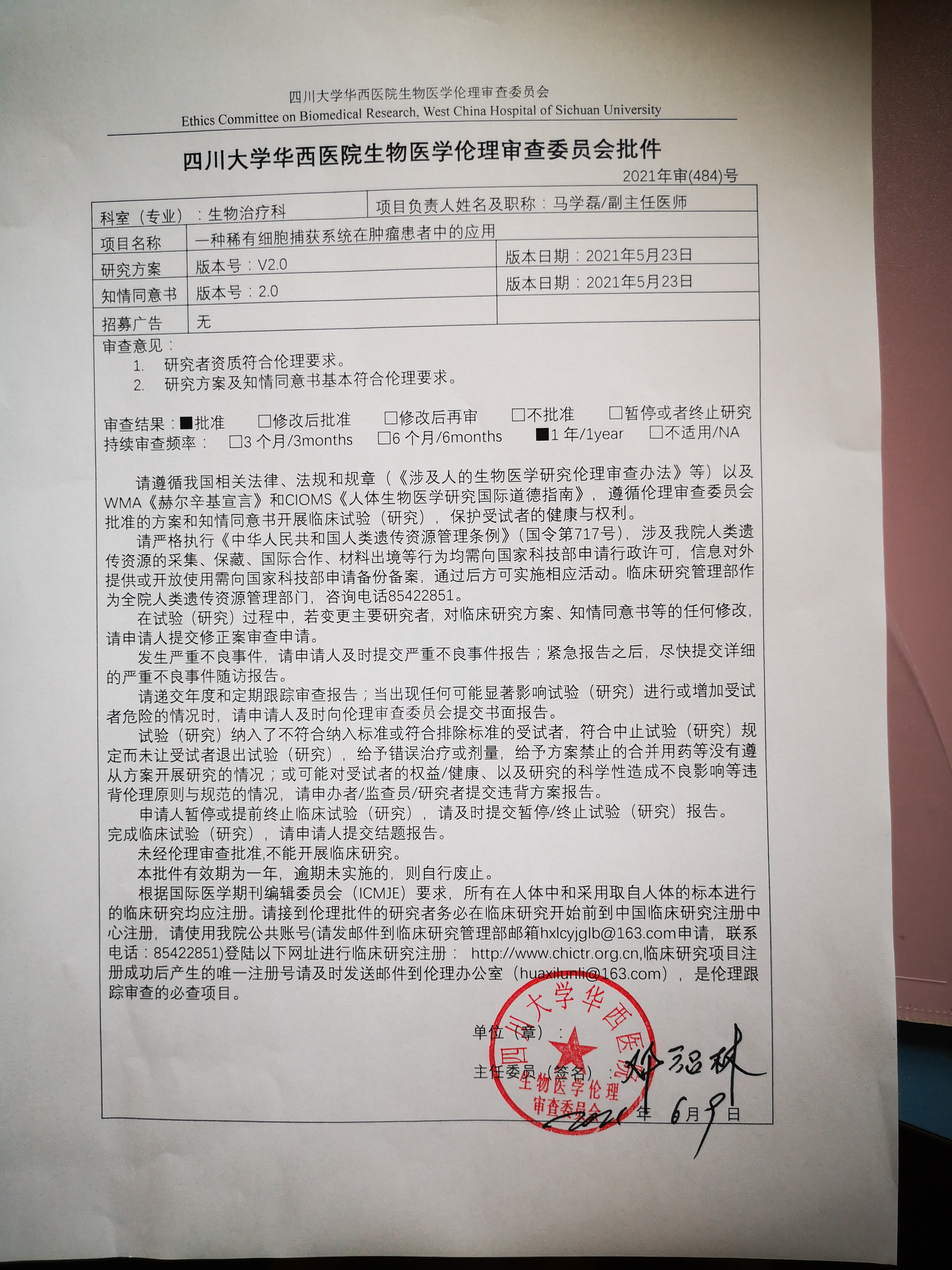

Supplement: Supplementary file 2 — Ethic Approval [file 41421_2024_646_MOESM2_ESM.jpg]
